# Examining the adoption and implementation of behavioral electronic health records by healthcare professionals based on the clinical adoption framework

**DOI:** 10.1186/s12911-022-01959-7

**Published:** 2022-08-08

**Authors:** Sooyoung Yoo, Kahyun Lim, Se Young Jung, Keehyuck Lee, Donghyun Lee, Seok Kim, Ho-Young Lee, Hee Hwang

**Affiliations:** 1 Office of eHealth Research and Businesses, Seoul National University Bundang Hospital, Seongnam, Republic of Korea; 2 Department of Family Medicine, Seoul National University Bundang Hospital, Seongnam, Republic of Korea; 3 Department of Education and Training, Seoul National University Bundang Hospital, Seongnam, Republic of Korea; 4 Department of Nuclear Medicine, Seoul National University Bundang Hospital, Seongnam, Republic of Korea; 5 Department of Pediatrics, Seoul National University Bundang Hospital, Seongnam, Republic of Korea

**Keywords:** Behavioral electronic health record (EHR) adoption, Clinical adoption framework, EHR implementation, Survey study, Structural equation analysis

## Abstract

**Background:**

While various quantitative studies based on the Unified Theory of Acceptance and Use of Technology (UTAUT) and Technology Acceptance Models (TAM) exist in the general medical sectors, just a few have been conducted in the behavioral sector; they have all been qualitative interview-based studies.

**Objective:**

The purpose of this study is to assess the adoption dimensions of a behavioral electronic health record (EHR) system for behavioral clinical professionals using a modified clinical adoption (CA) research model that incorporates a variety of micro, meso, and macro level factors.

**Methods:**

A questionnaire survey with quantitative analysis approach was used via purposive sampling method. We modified the existing CA framework to be suitable for evaluating the adoption of an EHR system by behavioral clinical professionals. We designed and verified questionnaires that fit into the dimensions of the CA framework. The survey was performed in five US behavioral hospitals, and the adoption factors were analyzed using a structural equation analysis.

**Results:**

We derived a total of seven dimensions, omitting those determined to be unsuitable for behavioral clinical specialists to respond to. We polled 409 behavioral clinical experts from five hospitals. As a result, the ease of use and organizational support had a substantial impact on the use of the behavioral EHR system. Although the findings were not statistically significant, information and service quality did appear to have an effect on the system's ease of use. The primary reported benefit of behavioral EHR system adoption was the capacity to swiftly locate information, work efficiently, and access patient information via a mobile app, which resulted in more time for better care. The primary downside, on the other hand, was an unhealthy reliance on the EHR system.

**Conclusions:**

We demonstrated in this study that the CA framework can be a useful tool for evaluating organizational and social elements in addition to the EHR system's system features. Not only the EHR system's simplicity of use, but also organizational support, should be considered for the effective implementation of the behavioral EHR system.

*Trial Registration*: The study was approved by the Institutional Review Board of Seoul National University Bundang Hospital (IRB No.: B-1904-534-301).

## Background

Since 2011, when the US government established the Health Information Technology for Economic and Clinical Health (HITECH) Act and the federal meaningful use program, usage of electronic health record (EHR) systems in general hospitals and clinics has been growing [1, 2]. The HITECH Act's purpose was to encourage the use of health information technology (IT). As a result, numerous research on the acceptance and implementation of EHR systems in various healthcare settings, except in the behavioral sector, have been done [3–5].

Behavioral health is a wide-reaching term that looks at how behaviors impact someone’s physical and mental health. In this study, behavioral health means mental health or psychiatry. Behavioral care settings and other settings differ in their language, roles, classifications, codes, data reporting requirements, and regulations [6]. As a result, earlier research in nonbehavioral hospitals may not be applicable equally to behavioral institutions. However, the setting of behavioral health has received little attention [7].

Recently, with the inclusion of behavioral health, which was formerly omitted from the HITECH Act's scope, research interest in adopting and implementing behavioral EHR systems has increased among healthcare experts [8]. Kruse et al. [9] conducted a review of 28 articles on EHR adoption in long-term care facilities and found improvement in the management of clinical documentation and quality outcomes although the impact on patient satisfaction, physician satisfaction, and productivity were not well shown. Additionally, Sadoughi et al. [10] conducted a meta-analysis of 16 articles and addressed that perceived ease of use, intention, attitude, performance, and social influence all play a significant role in EHR system adoption. As evaluation methods, interviews, questionnaires, and usability measurement methods were mainly used, and it was reported that the focus was on the use of multi-method [10].

In terms of models utilized in EHR system adoption research, the technology acceptance model (TAM) and the unified theory of acceptance and use of technology (UTAUT) have been the most often used [11–13]. These models are extensively utilized because they are relatively simple to use in conducting EHR adoption research; nonetheless, some researchers have warned that their breadth may be too limited. Alternatively, the clinical adoption (CA) framework has arisen as a model for assessing technology adoption in the healthcare sector [14]. The CA framework has three conceptual views of eHealth adoption for clinical professionals in different settings—the micro, meso, and macro level. The micro level addresses the quality of the information, systems, and services associated with an eHealth system. The meso level addresses the people, organization, and implementation dimensions that have a direct effect on the micro level eHealth adoption by clinical professionals. The macro level addresses healthcare governance, standards, funding, and societal trends as the environmental factors. The CA framework has an advantage over other models in that it not only defines system-level variables in detail, but also analyses meso and macro levels, such as institutional leadership, policy, and socioeconomic trends. However, because the model is abstract and does not contain data for questionnaires, the research on its application has been limited [15].

As a result, we refined the CA-based research model for evaluating a behavioral EHR system to address the shortcomings of previous research models. Consultations with behavioural clinical professionals and a pre-survey were used to verify it. Following that, we ran a structured survey to assess adoption of a behavioral EHR system and deduce variables associated with effective implementation.

## Methods

### Research model design

Based on the CA framework, we designed a research theory model for evaluating EHR adoption by behavioral medical professionals that addresses the drawbacks of TAM and UTAUT (i.e., models that have been frequently used in existing healthcare environments). However, the CA framework does not provide actual question items. Accordingly, through literature review, we collected all the verified questionnaire items related to each factor of a CA framework. A set of survey questions were then constructed for each dimension of the CA framework with reference to existing studies. We collected 161 questions with 11 dimensions. Questions were selected by considering the definition of each dimension described in the CA framework, and the subdimensions for each dimension were classified, as well. A physician, a medical informatics professor, and three researchers met weekly for three months to search, organize, and classify question items. After classifying the question items into dimensions, about two to four representative questions were chosen for each dimension to build a draft survey questionnaire, avoiding those with overlapping or confusing meanings. Finally, 52 questions for the general participants remained.

After developing draft survey questionnaires, a preliminary survey was conducted with 136 hospital employees responsible for EHR training at a study site behavioural hospital. The preliminary study's objective was to enhance the research model and question items and to ensure the questionnaire's internal consistency. To ensure the responses were reliable, we created two or three similar questions for each dimension or sub-dimension. Respondents indicated that certain of the CA framework's elements were inappropriate for behavioral clinical specialists. These questions were eliminated following consultation with three corporate staffs (one C-level and two nurses) participating in the EHR implementation of the behavioral hospitals. Accordingly, the macro level of healthcare standards, regulation, and governance, as well as funding and incentive elements, was omitted. People and implementation were omitted at the meso level. The new questionnaire was subsequently edited to incorporate feedback from three medical staff members from behavioural hospitals regarding their ability to communicate in English, including tone and expression. To ensure truthful responses, the sequence of the questions was randomly mixed.

**Fig. 1:**
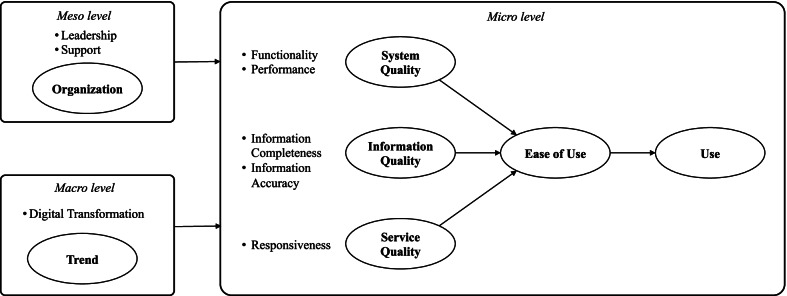
Research Model. Research Hypotheses: H1: System quality will have a positive impact on ease of use. H2: Information quality will have a positive effect on ease of use. H3: Service quality will have a positive effect on ease of use. H4: The ease of use will have a positive impact on use. H5: The trend will have a positive effect on use. H6: The organizational factor will have a positive impact on use

A preliminary survey was conducted at the Aurora Glendale hospital and Aurora Tempe hospital in Arizona. After the survey was administered, a factor analysis was performed on the results. When the results of the factor analysis indicated that a question item did not load on the same factor as the other items within the dimension, the item was removed to facilitate subsequent analysis, such as reliability analysis and structural equation modeling (SEM). For the final questionnaire, in addition to multiple-choice questions, an open-ended question asking for opinions on positive and negative effects when using the EHR system was added to collect the free opinions of the survey participants. Figure 1 shows the final research model and hypotheses.

### Study sites

This study was conducted at five Aurora behavioral hospitals that are under the same parent corporation in Arizona and San Francisco in the United States, namely, Aurora Charter Oak (ACO), Aurora Glendale (AGD), Aurora Tempe (ATP), Aurora Vista Del Mar (AVM), Aurora Reno (ARN). In 2017, the hospital cooperation decided to implement a behavioral EHR system by customizing by adding behavioral features to a commercial EHR system.

### Survey

The survey was conducted from September 22, 2019, to November 07, 2019, at the five study sites. With the permission of the director of the hospitals and support of the head nurse, we set up an online survey by using the computers of each hospital’s training room. Two researchers manned the training room at each hospital to enable every member of a shift to participate in the survey. Medical staff members participated before, after, or during work. The survey took approximately 10–15 min, and a USD 10 Amazon gift card was provided to all participants who completed the survey. The subjects participated in the survey only for those who voluntarily expressed their consent after receiving a sufficient explanation of the background and purpose of the study along with an explanation of their rights. Since a research conducted by Seoul National University Bundang Hospital (SNUBH)-affiliated researchers should be deliberated by SNUBH’s institutional review board (IRB) for conducting research in the US, the study was approved by the institutional review board of SNUBH (IRB No.: B-1904–534-301). A written consent was exempted because this study, conducted as an online survey, did not contain sensitive personally identifiable information and there was no disadvantage or risk to the subject's mental and physical health. Thus, the informed consent was obtained from all the individuals by checking the online consent of the participants to participate in the online survey instead of the written consent. This study was also conducted in collaboration with Aurora behavioral health hospitals, that supported this research to SNUBH, which developed the base version of the EHR solution they introduced.

### Data analysis

For the analysis of survey results, we removed insincere responses from the examination of survey findings, such as those supplied by respondents who answered the same way to all questions. Similarly, we omitted replies from nonclinical professional respondents because this study focused on the system's intended users, namely clinical professionals such as physicians and nurses. Following that, we used SPSS to conduct statistical analyses, including factor analysis and reliability analysis. Finally, SEM was used to evaluate the structural correlations between dimensions using AMOS26. The structural equation analysis was chosen to explore the causal relationships among latent variables of interest that are not directly observable and simultaneously analyze the influence relationship between multiple variables.

Regarding the responses to open-end questions about positive and negative effects when using the EHR system, researchers and medical information professors classified, and reviewed respondents' opinions based on keywords for free-text opinions. Opinions on the positive effect were mainly classified into efficiency, collaboration, and safety. On the other hand, opinions on negative effects were divided into opinions in various areas such as speed, inconvenience, record, inefficiency, dependency, and alert. The number of opinions according to the relevant classification was arranged to summarize the frequent opinions.

## Results

### Survey questionnaire

The final questionnaire is shown in Table 1, which was developed following a literature study, a pre-survey, external consultations, and internal review sessions. An open-ended question was also included at the end of the questionnaire to elicit opinions that were not reflected in the multiple-choice responses. The majority of respondents provided extensive responses, and we classified and evaluated the survey comments using qualitative methods of analysis.

**Table 1:** Survey items

Questionnaire	References^a^
System quality-functionality	
FT1. The system is effective in documenting patient clinical notes	[16]
FT2. The system provides useful information for psychiatric clinical decision making	[17]
System quality-performance	
PF1. The system is reliable in its performance	[17]
PF2. The system speed is good enough for my day-to-day tasks	[18]
Information quality-information completeness	
IC1. The information in the system provides sufficient breadth and depth for my daily practice	[19]
IC2. I can accomplish all my regular tasks with the information found in the system	[19]
IC3. The system includes all the information required for my daily practice	[19]
Information quality-information accuracy	
AC1. The information I get from the system is accurate	[20]
AC2. The information in the system is accurate	[16]
Service quality-responsiveness	
RE1. When there is a problem with the system, the support team resolves my problems quickly	[21]
RE2. The system provider gives me prompt service	[21]
Ease of use	
EA1. The system is easy to learn	[20]
EA2. It is easy to master the functions in the system	[22]
Use	
US1. I use the system as part of my day-to-day tasks	[23]
US2. I cannot accomplish my tasks without having to use the system	[23]
Organization	
OR1. The hospital’s leadership encourages the use of the system	[24]
OR2. The hospital provides enough the system training programs	[25]
OR3. The hospital strongly supports digital innovation	[26]
Trend	
TR1. How far has the current medical industry shifted from a paper to digital transformation?	[26]
TR2. Do you consider digital transformation as a key business driver in your hospital?	[26]
Net Benefit	
NB1. The system helps to improve patient safety	[15]
NB2. The system helps to improve continuity of care	[15]
NB3. The system helps to improve quality of care	[15]
NB4. The system helps to improve the coordination of care for the patient	[15]
NB5. The system helps to improve my efficiency in completing tasks	[15]
NB6. The system helps to improve our staff’s ability to access patient information	[15]
NB7. The system helps to improve staff satisfaction	[15]
NB8. The system helps to improve patient satisfaction	[15]

^a^Reference number. In the actual survey, the name of the EHR system was used in the system phrase

### Participant characteristics

The survey elicited responses from 409 clinical experts at five behavioral hospitals. We removed 29 respondents' responses during data preprocessing because they were not healthcare professionals or did not use the EHR system for work, such as accounting or business office workers. There were 1,699 clinical professionals employed in the five institutions, of whom 380 responded to the survey, for a response rate of 22.4 percent. The response rate for ACO was 30.4 percent, AGD was 21.2 percent, ATP was 13.3 percent, AVM was 26.7 percent, and ARN was 11.8 percent. Additionally, 14 respondents who replied the same way for 42 questions and 9 respondents who scored the negative and positive questions the same way were eliminated, leaving 346 responses for analysis.

Table 2 summarises the characteristics of all subjects. 77.75 percent of responders were clinical professionals under the age of 50. 22.25 percent of respondents over the age of 50 had a low level of IT literacy. Additionally, 85% of respondents have attended EHR training, and approximately 91% of those who did so stated that it was not difficult.

**Table 2:** Participant characteristics

Characteristics	N	Percent (%)
Working hospital		
ACO	126	36.42
AGD	70	20.23
ATP	73	21.10
AVM	52	15.03
ARN	25	7.23
Gender		
Female	261	75.43
Male	85	24.57
Age (Years)		
~ 30	83	23.99
31–40	111	32.08
41–50	75	21.68
51–60	56	16.18
61–70	20	5.78
≥ 71	1	0.29
Occupation		
Adjunctive therapist	9	2.6
BHT/MHT	47	13.58
Case Manager	73	21.10
Counselor	3	0.87
Inpatient RN	91	26.30
Intake RN	35	10.12
Outpatient RN	10	2.89
LVN/LPT	18	5.20
Pharmacist	8	2.31
Psychiatrist	2	0.58
Psychologist	2	0.58
Internist	2	0.58
UR Specialist	19	5.49
Years of Experience		
~ 1	34	9.83
~ 5	128	36.99
~ 10	76	21.97
~ 20	67	19.36
~ 30	30	8.67
~ 40	11	3.18
EHR experience		
None	103	29.77
1	86	24.86
2	73	21.10
More than 3	84	24.28
Training participation		
Yes	295	85.26
No	51	14.74
Training experience		
Easy	81	27.46
Adequate	188	63.73
Difficult	26	8.81

### Structural equation modeling

**Fig. 2:**
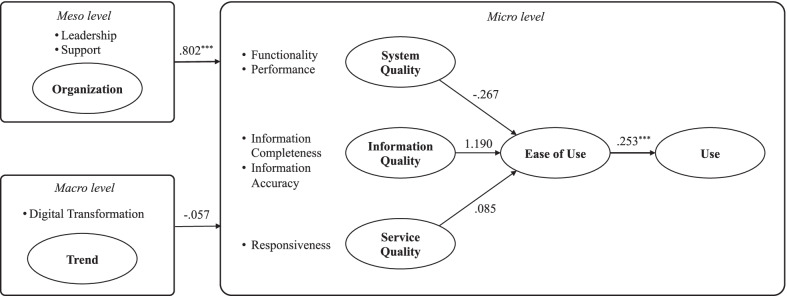
Results of SEM analysis

In Fig. 2, the result of the SEM is depicted. The ease of use of the behavioral EHR system and organizational support both had a positive influence on the use of the system.

### Model fit

The chi-square value for model fit was 1318.174, with 183 degrees of freedom, and the *P*-value was < 0.001, which was less than 0.05. The goodness-of-fit index (GFI) was 0.732 (0–1.0 recommended), and the root mean square residual was 0.287 (0–0.05 recommended), both of which were slightly higher than recommended values. The root mean square error of approximation was 0.133 (0.05–0.08 recommended). The normed fit index, comparative fit index, Tucker–Lewis index, and adjusted GFI were all somewhat lower than the recommended values of 0.725 (0.9–1.0 recommended), 0.752 (0.9–1.0 recommended), 0.716 (0.9–1.0 recommended), and 0.661 (0.9–1.0 recommended), respectively.

As illustrated in Table 3, H4 (Ease of Use Use) and H6 (Organization Use) were statistically significant among the ten hypotheses generated in this study. Additionally, none of the hypotheses H1 (System Quality Ease of Use), H2 (Information Quality Ease of Use), H3 (Service Quality Ease of Use), and H5 (Trend Use) were strongly supported (p-value > 0.05). It seems that System Quality, Information Quality, and Service Quality comprehensively affect Ease of Use rather than one specific factor significantly affecting ease of use.

**Table 3:** Path coefficient results

H	Path			Estimate	S.E	C.R	*P*
H1	System quality	→	Ease of use	− 0.267	2.605	− 0.103	0.918
H2	Info. quality	→	Ease of use	1.190	2.222	0.536	0.592
H3	Service quality	→	Ease of use	0.085	0.621	0.137	0.891
H4	Organization	→	Use	0.802	0.086	9.307	***
H5	Trend	→	Use	− 0.057	0.073	− 0.776	0.438
H6	Ease of Use	→	Use	0.253	0.048	5.258	***

***P < 0.001

*S.E*. standard error, *C.R*. composite reliability

### Results for net benefit dimension

**Fig. 3:**
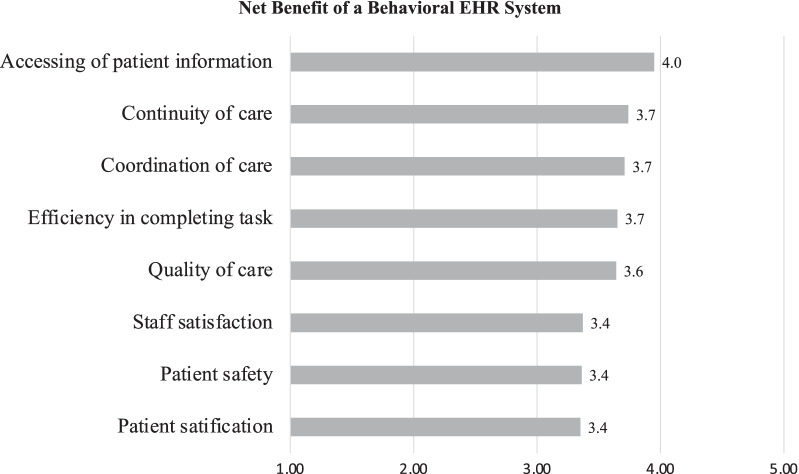
Net benefit survey result

On a five-point scale, the average score for all eight questions in the net benefit dimension was 3.6. Among the eight questions of (i) accessing of patient information, (ii) continuity of care, (iii) care coordination, (iv) work efficiency, (v) quality of care, (vi) staff satisfaction, (vii) patient safety, and (viii) patient satisfaction, clinical professionals in behavioural hospitals reported that the EHR system is more than moderately helpful in improving accessing of patient information, continuity of care, and care coordination (see Fig. 3).

### Positive and negative opinions of EHR

Positive responses to open questions indicated that work efficiency increased as a result of the EHR system. They reported that using the copy and paste tool resulted in faster searching for patient information and less time spent charting. Establishing a master treatment plan was becoming easier as a result of the departments' excellent collaboration. Therefore, staff members will be able to spend more time with patients. Additionally, they valued the option of accessing the EHR via a mobile application. They asserted that the EHR system delivers sufficient and suitable data for behavioral activities. Moreover, the user-friendly design and double-check alert feature were complimented.

The majority of negative evaluations concerned system stability. Due to the network infrastructure of the institutions, the EHR system occasionally froze or slowed down. One of the concerns was an over reliance on the EHR system.

In certain instances, there were divergent views. According to some, the EHR supplied little information, while others claimed it provided much data. Some noted a deficiency in the number of notifications and obligatory fields, while others claimed an overabundance of alerts and mandatory data.

## Discussion

### Principal findings

We examined the adoption of a behavioral EHR system from the perspective of behavioral clinical professionals in this article. We utilized a research theory model that addresses the TAM and UTAUT models' inadequacies, namely the CA framework. The CA framework takes into account not only system-level features, but also broader ones such as institutional leadership, policy, and socioeconomic trends. We derived question items and dimensions from the literature and expert consultations for applying the CA framework. After the structured survey in five US behavioral hospitals, we analyzed the results using structural equation analysis.

Our study found that organizational support was the most critical factor in ensuring the effective implementation of behavioral EHRs; nevertheless, ease of use was also regarded critical. For organizational support, it was critical to provide education on how to utilize the EHR system, to invest at the level of organizational leadership, and to motivate. Contrary to our expectations, neither information nor service quality were noteworthy. Additionally, the open questions revealed that the majority of respondents agreed on the benefits of EHR, and we discovered that there were more complaints about system freezing caused by network issues than system issues.

Given that behavioral EHR has been a relatively isolated subject, there are unlikely to be many medical staff members who have worked with EHR in behavioral hospitals. About 55% of interviewees had no prior involvement with an EHR system. Nonetheless, a consensus regarding its merits has developed as a result of the societal circumstances. To minimize the barrier to entry, the system should be as simple to configure as possible. Sufficient education and motivating courses are also significantly recommended for continued professional growth at the institutional level. Additionally, because behavioral institutions are frequently located outside of city centers and the network may be unstable, a thorough prior examination of the network's resilience is essential to avoid exhausting users.

### Limitations

This study utilized a single behavioral EHR system across five institutions. There are just a few behavioral hospitals that have previously implemented an EHR system, and securing their cooperation proved challenging. The fact that the research was done exclusively on connected hospitals utilizing a single vendor's EHR system significantly limited the study's ability to adequately reflect the characteristics of varied organizations and systems. To address these constraints, this study surveyed diverse occupational groups in five regions across the United States.

Additionally, opinions of non-clinical professionals in executive C-level or administration departments were not gathered. While clinical professionals are critical in implementing EHR systems, the diverse range of occupations undoubtedly influences decision-making. Additionally, as implied by the original CA framework, a lack of consideration for more diverse factors such as cost, law, personnel, implementation, and motivation was evident. Additional research is necessary to investigate these aspects, and we anticipate that this work will serve as the foundation for future research.

### Comparison with Prior Work

Sadoughi et al. [10] discovered that the primary factors impacting EHR implementation are the system's ease of use and performance. Additionally, Kruse et al. [9] highlighted technical support, initial cost, training, and workflow issues as significant determinants of EHR system implementation. Technical support is comparable to "responsiveness," a sub-dimension of the "service quality" dimension examined in this study, whereas initial cost and training are comparable to the "organization" dimension examined in this study.

According to subjective responses in the survey, the most critical functions of the EHR system were its suitability for behavioral workflow, the provision of appropriate information to aid in behavioral decision making, and the convenience of the clinical note documentation features that allow for free text charting. Additionally, it was critical to examine aspects at the meso and macro levels in our study, such as the impact of the societal trend on the organization and its leadership, as well as support beyond the system level.

## Conclusions

This study evaluated the introduction of a behavioral EHR system for behavioral clinical professionals. Based on the CA framework, we devised a model that considered the organizational and social atmosphere of the system beyond the system level. The results indicate that ease of use and organizational support positively influenced the use of the behavioral EHR system. In terms of organizational support, for the successful introduction of the behavioral EHR system, policy efforts are required to form a vision and culture for digital innovation within the organization through strong leadership. Additionally, in order to provide end-users with a sufficient system training program and motivate their participation, it will be helpful to nurture power users within the organization and to conduct regular educational activities through them. In terms of the EHR system, in particular, the usability evaluation factor should be considered important when introducing the behavioral EHR system.

## Data Availability

The data used in this study cannot be shared owing to a policy of the institutional review board of SNUBH. The first author (yoosoo0@snubh.org) can be contacted regarding a request on data and materials.
